# Effect of dietary intervention on serum lignan levels in pregnant women - a controlled trial

**DOI:** 10.1186/1742-4755-7-26

**Published:** 2010-10-08

**Authors:** Riitta Luoto, Elham Kharazmi, Niina M Saarinen, Annika I Smeds, Sari Mäkelä, Mahdi Fallah, Jani Raitanen, Leena Hilakivi-Clarke

**Affiliations:** 1 UKK Institute for Health Promotion, Tampere, Finland; 2 Tampere University Central Hospital, Tampere, Finland; 3 University of Turku, Turku, Finland; 4 Åbo Akademi University, Laboratory of Organic Chemistry, Turku, Finland; 5 Tampere School of Public Health, University of Tampere, Tampere, Finland; 6 Georgetown University, Washington DC, USA; 7 National Institute for Health and Welfare, Helsinki, Finland

## Abstract

**Background:**

Mother's diet during pregnancy is important, since plant lignans and their metabolites, converted by the intestinal microflora to enterolignans, are proposed to possess multiple health benefits. Aim of our study was to investigate whether a dietary intervention affects lignan concentrations in the serum of pregnant women.

**Methods:**

A controlled dietary intervention trial including 105 first-time pregnant women was conducted in three intervention and three control maternity health clinics. The intervention included individual counseling on diet and on physical activity, while the controls received conventional care. Blood samples were collected on gestation weeks 8-9 (baseline) and 36-37 (end of intervention). The serum levels of the plant lignans 7-hydroxymatairesinol, secoisolariciresinol, matairesinol, lariciresinol, cyclolariciresinol, and pinoresinol, and of the enterolignans 7-hydroxyenterolactone, enterodiol, and enterolactone, were measured using a validated method.

**Results:**

The baseline levels of enterolactone, enterodiol and the sum of lignans were higher in the control group, whereas at the end of the trial their levels were higher in the intervention group. The adjusted mean differences between the baseline and end of the intervention for enterolactone and the total lignan intake were 1.6 ng/ml (p = 0.018, 95% CI 1.1-2.3) and 1.4 ng/mg (p = 0.08, 95% CI 1.0-1.9) higher in the intervention group than in the controls. Further adjustment for dietary components did not change these associations.

**Conclusion:**

The dietary intervention was successful in increasing the intake of lignan-rich food products, the fiber consumption and consequently the plasma levels of lignans in pregnant women.

**Trial registration:**

ISRCTN21512277, http://www.isrctn.org

## Introduction

During pregnancy, the fetus is exposed to multiple biologically active compounds that originate from the maternal diet. Some of these may affect offspring's later health. Dietary phytoestrogens are examples of such compounds, as they are found in the amniotic fluid and the umbilical cord blood [1]. In the Western diet, phytoestrogens consist mainly of plant lignans, which are a large group of fiber-associated phenolic compounds, widely distributed in edible plants, e.g., seeds, whole grains, vegetables, fruits and berries [2,3]. Significant dietary sources of fiber-associated phenolic compounds are beverages such as coffee, tea, and red wine [2,3]. Many of the ingested plant lignans in food are converted by intestinal microbiota to enterolignans, e.g., enterodiol (ED) and enterolactone (ENL) [4]. Enterolignans undergo enterohepatic circulation [5] and are thereafter found in a number of tissues [6].

Dietary lignans have been portrayed as health-promoting agents in many epidemiological studies, which show an inverse association between plant lignan intake or serum/urine enterolignan concentrations and chronic Western life-style diseases, including breast and colon cancer, and cardiovascular diseases [7]. Furthermore, chemopreventive actions of lignans against mammary cancer have been demonstrated in experimental models [8,9]. They are potentially mediated through mechanisms such as modulation of estrogen action and by possessing anti-angiogenic, pro-apoptotic, and anti-oxidant properties [9].

Several studies indicate that supplementation of a habitual diet with lignan-rich foods can affect circulating enterolignan concentrations in women [7]. However, there are only scarce data available on serum lignan concentrations of pregnant women [10,11], and there are no studies available that have investigated whether a dietary intervention towards "a healthier diet" through dietary counselling would influence enterolignans and other commonly occurring plant lignans in pregnant mothers. Main aim of the intervention study was to find out, whether counselling on physical activity, diet and weight development have effect on pregnancy weight development [12,13]. The goal of dietary counseling was to modify the diet of first-time pregnant women according to the current nutritional recommendations. Aim of this study is to report changes in lignan concentrations among experimental groups. To our knowledge, this is the first study to determine whether changes in the habitual diet can affect serum lignan concentrations of pregnant women.

## Methods

### Setting and study design

Each municipality in Finland is responsible for arranging maternity health-care services for its residents, and these are covered by public tax revenue. Almost all (99.7%) pregnant women attend public maternity clinics [14]. The present study was conducted at six volunteering clinics in the city of Tampere and the town of Hämeenlinna, both located in the southern Finland. The study was approved by the Ethics Committee of the Pirkanmaa Hospital District. A flow chart of the study is shown in additional files, additional file 1.

In the Finnish maternity health-care system, pregnant women are recommended to visit 11-15 times a nurse and three times to a physician. This study was implemented during two of these routine visits to nurses at gestation weeks 8-9 (baseline) and 36-37 (end of intervention).

### Participants

The participants were pregnant women with no earlier deliveries. Women who were younger than 18 years of age, or suffered from type I or type II diabetes mellitus, twin pregnancy, physical disability that prevented exercising or had otherwise problematic pregnancy, substance abuse, treatment or clinical history of any psychiatric illness, were unable to speak Finnish, or intended to change residence within three months, were excluded from the study. The nurses recruited the participants when they enrolled for their first clinic visit at the beginning of their pregnancy, usually by phone. Forty-nine women in the intervention clinics and 56 women in the control clinics gave informed consent for participation. Recruitment took place between August 2004 and January 2005.

The participants visiting the control clinics received the standard maternity care. The intervention group received recommendations for gestational weight gain and physical activity counseling, the details of which are presented elsewhere [12,13].

For the dietary counseling, the following dietary objectives were set for each participant to achieve or maintain: 1) to follow a regular meal pattern, emphasizing the importance of breakfast and > = 1 hot meal every day, 2) to eat at least five portions (400 g) per day, in total, of different kinds of vegetables, fruits and berries, 3) to consume preferentially high-fibre bread (> = 5 g fibre/100 g), and 4) to restrict the intake of high-sugar snacks to < = 1 portion per day (e.g., 50 g sweets, one pastry, once piece of cake, two biscuits, 2 dl of ice cream or a glass of soft drink). The dietary counselling consisted of one primary counselling session (allocated time 20-30 min) at the visit during gestation weeks 16-18, and three booster sessions (allocated time 10 min) until gestation week 37.

### Data collection

The pre-pregnancy weight and height were self-reported, whereas weight development during the pregnancy was based on measurement by nurses in maternity centers. The baseline questionnaire, including questions on background, lifestyle and dietary intake (a 57-item food frequency questionnaire), was completed before the first visit (at gestation week 8 or 9). The second follow-up questionnaire was completed at the end of the study, on gestation week 37. Similar FFQ was filled by both groups. The baseline dietary information was based on the diet during the month before the pregnancy and the follow-up information on the diet during the month before the 26 to 28 pregnancy week's visit.

### Lignan measurements

The plant lignans 7-hydroxymatairesinol, secoisolariciresinol, matairesinol, lariciresinol, cyclolariciresinol, and pinoresinol, and the enterolignans 7-hydroxyenterolactone, enterodiol, and enterolactone were quantified in the serum using a previously developed and validated high-performance liquid chromatography-tandem mass spectrometric (HPLC-MS/MS) method [15]. Briefly, the method included enzymatic hydrolysis, solid-phase extraction, and analysis using a triple quadrupole mass spectrometer with electrospray ionisation in the multiple-reaction monitoring mode. The serum lignans were quantified using deuterated matairesinol or enterolactone as internal standards. Pinoresinol, which was not included in the validated method, was added among the analytes. The MS/MS transition monitored for this compound was the deprotonated molecular ion to the predominant fragment ion of *m/z *151, and deuterated matairesinol was used as internal standard.

### Statistical methods

Baseline information on age, pre-pregnancy BMI, weight gain during pregnancy, education level and smoking status before and during pregnancy were reported by both in the intervention and the control groups. Education was self-reported and categorized as basic or secondary education, polytechnic or university training. Smoking was inquired both before (non-smokers or quitters; daily or occasional smokers) and during pregnancy (occasional smoking or non-smoking). These variables were later included, when necessary, as confounding factors in the multivariable analyses. In all statistical analyses, p < 0.05 was used as the level of statistical significance.

The group differences of lignans at the end point of both follow-ups adjusted for the baseline level of lignans and other possible confounders were examined by the linear regression model. The original lignan values were skewed, and were transformed by using the logarithm to enable statistical analysis requiring normally distributed variables. The results at the end point of each follow-up are illustrated as a group difference calculated using antilogs of mean differences of log-transformed variables, and presented with 95% CI.

In the regression model, the analyses included baseline level of lignans, age, BMI, education, smoking and change in consumption of vegetables and fruits. Further adjustment was performed for dietary components that affect lignan intake or are thought to intervene with lignan metabolism. These were coffee, tea, other drinks, butter, potato, peas, milk products, pork, beef, and sugar. Since these dietary variables were not significant, they were dropped out from the backward stepwise regression model.

## Results

Women in the intervention group were younger, less educated, and were more often smokers, and they had a higher pre-pregnancy weight and BMI on average than the women in the control group (Table 1).

**Table 1:** Background variables in control and intervention groups

	Control	Intervention	Control		Intervention		
				
Characteristic	N	N	mean	SD	mean	SD	P-value
Pre-pregnancy age	56	49	28.8	4.1	27.4	4.6	0.105^1^
							
Pre-pregnancy BMI	56	49	22.3	2.1	23.6	4.0	0.036^1^
							
Change in fiber (g/day)	53	49	-0.2	6.3	2.7	6.5	0.0216^1^
							
Smoking during pregnancy			%		%		0.260^2^
Occasionally	3	0	3.1		0.0		
Not at all	53	41	54.6		42.3		
							
Pre-pregnancy smoking			%		%		0.065^3^
Non-smoker/quitted	46	32	45.1		31.4		
Daily/occasionally	9	15	8.8		14.7		
							
Education			%		%		0.062^3^
Basic/secondary level	20	27	19.4		26.4		
Polytechnic school	12	9	11.8		8.9		
University	24	11	23.7		10.9		

1) T-test

2) Fisher's Exact Test

3) Chi-square

Enterolactone was detectable in almost all the samples (Table 2). Other lignans, such as hydoxyenterolactone, matairesinol, and lariciresinol were detected only in very few women. Among the women in the intervention group, the concentration of almost all serum lignans increased during intervention, while among the controls the serum concentrations of lignans other than enterolactone either did not change or they were reduced. The baseline levels of enterolactone, enterodiol and the sum of the lignans were higher in the control group than in the intervention group. However, as a result of the intervention, the levels of lignans in the intervention group increased to a significantly higher level than among the controls (Table 2).

**Table 2:** Percentiles (%) of women exhibiting different lignans, average concentrations and concentration range (ng/ml) of individual lignans in the control and intervention groups at the baseline and at the end of intervention.

	Baseline (gestational week 8 or 9)			End (gestational week 36 or 37)		
	
Control*	Detected in number (%) of samples	Mean ng/ml (SD)	Range (nd = not detected)	Positive samples	Mean ng/ml (SD)	Range
ENL	53	0.46 (0.68)	nd-4.4	52	0.48 (0.38)	0.03-2.4
HENL	14	0.03 (0.024)	nd-0.09	17	0.02 (0.05)	0-0.18
SECO	4	0.22 (0.077)	0.15-0.33	12	0.12 (0.09)	0-0.3
MR	39	0.003 (0.01)	nd-0.07	49	0.003 (0.01)	0-0.05
END	28	0.22 (0.34)	nd-1.8	32	0.22 (0.31)	0-1.45
LAR	9	0.14 (0.1)	nd-0.29	3	0.01 (0.02)	0-0.04
CLAR	10	0.13 (0.23)	nd-0.58	15	0.14 (0.12)	0-0.43
PINO	1	0.58 (0)	0.58-0.58	4	0.25 (0.27)	0-0.61
Sum of all lignans	56	0.63 (0.82)	nd-4.61	56	0.66 (0.65)	0-3.48
Intervention*						
ENL	49	0.34 (0.39)	nd-2.2	49	0.85 (1.4)	0.09-7.4
HENL	10	0.03 (0.02)	nd-0.064	15	0	0
SECO	2	0.10 (0.09)	0.04-0.17	10	0.16 (0.15)	0.015-0.5
MR	33	0.004 (0.02)	nd-0.13	45	0.004 (0.01)	0-0.07
END	32	0.12 (0.12)	nd-0.33	32	0.19 (0.20)	0-0.82
LAR	7	0.03 (0.05)	nd-0.11	2	0.05 (0.07)	0-0.1
CLAR	9	0.10 (0.16)	nd-0.48	9	0.04 (0.07)	0-0.2
PINO	3	0.58 (0.26)	0.30-0.81	4	0.21 (0.15)	0-0.33
Sum of all lignans	49	0.49(0.48)	0.01-2.25	49	1.03 (1.56)	0.14-8.16

*ENL = enterolactone, HENL = 7-hydroxyenterolactone, SECO = secoisolariciresinol, MR = matairesinol, END = enterodiol, LAR = lariciresinol, CLAR = cyclolariciresinol, PINO = pinoresinol

Group differences were analyzed taking into account baseline lignans, age, BMI, education and smoking. The adjusted mean difference for enterolactone at the end of the intervention when compared to the baseline was 1.6 ng/ml higher in the intervention group than in the controls (p = 0.018; 95% CI: 1.1-2.3, adjusted for lignan at baseline, age, BMI, education and smoking). Similar results were found for enterolignan (P = 0.021; 95% CI: 1.1-2.2). The mean adjusted difference between baseline and end for the sum of lignans was 1.4 ng/ml (P = 0.083; 95% CI: 1.0-1.9) higher in intervention group than in the controls (Table 3). Group differences were additionally analyzed by including change in consumption of vegetables and fruits, meat, and bread in the backward stepwise selection regression model. Only the baseline level of enterolignans, the change in vegetable and fruit consumption, and education were significantly correlated with the group differences (Table 3).

**Table 3:** Group differences between enterolactone, enterolignan and the sum of lignans (ng/ml) at the end of the intervention*.

	Enterolactone			Enterolignan			Sum of lignans		
	
	coeff	p	95% CI	coeff	p	95% CI	coeff	p	95% CI
Intervention vs control,	1.6	0.018	1.1-2.3	1.5	0.02	1.1-2.2	1.4	0.08	1.0-1.9
Lignans, baseline	1.61	0.01	1.16-2.24	1.42	0.00	1.26-1.60	1.29	0.04	1.01-1.29
Change in consumption of vegetables and fruits	1.14	0.04	1.01-1.30	1.53	0.02	1.08-2.16	1.14	0.03	1.01-1.14
University vs. basic/secondary school	1.83	0.01	1.20-2.81	1.62	0.02	1.09-2.41	1.81	0.00	1.23-1.81
Polytechnic vs. basic or secondary school	2.37	0.00	1.45-3.89	0.37	0.00	0.27-0.50	2.41	0.00	1.54-2.41

*All results in the table have been adjusted for baseline level of lignans, age, BMI, education, smoking, change in consumption of vegetables and fruits.

## Discussion

The results obtained in our controlled trial involving pregnant women indicate that the serum levels of enterolignans at the end of the intervention period were significantly higher in the intervention group than in the controls. This is consistent with our earlier results showing that intake of vegetables, fruits, berries and fiber - all of which contain lignans - is significantly higher in the pregnant intervention group than in the controls [16].

Strengths of our study include experimental, controlled design and exact measurements of lignans based on blood samples and nutrition based on large food frequency questionnaires. Other strengths of the study were that the participation rate to the intervention was excellent, as only a few visits were missed [13]. To date, similar findings have not been reported in any experimental study among pregnant women and thus our findings are unique.

The concentrations of enterolactone in serum are associated with consumption of lignan-containing foods and constipation [17], and urinary lignan excretion is positively associated with dietary fiber intake, as well as with diets that are high in fiber and carbohydrates and lower in fat. Coffee and tea also affect plasma concentrations of enterolactone [18]. Our results show that the baseline (pregnancy weeks 8-9) consumption of lignan-rich ingredients, such as vegetables and fruits, and the level of education are the main confounders on the adjusted mean difference in enterolactone levels. Well-educated individuals are known to consume more fruits, vegetables and fiber than less educated individuals [19], and this likely caused the association between education and enterolactone levels during pregnancy observed in this study. However, dietary intake or education appeared to explain only a small proportion of our results. The role of gut microflora in the metabolism of lignans is known to be important, and future studies are needed to determine the bioavailability and absorption rate of lignans in pregnant women consuming diets high in vegetables, fruits and fiber.

The results reported here are based on a pilot study designed to test the intervention protocol for a larger study. The lack of randomization of the maternity clinics was probably the most important limitation of the present study. The baseline differences in several factors between the intervention and control groups might have been smaller had we been able to randomize the clinics. On the other hand, the women themselves do not make choices between clinics, because they are directed to maternal centers based on their living area. Thus, non-randomization is not as large bias as in the case of randomization by patient basis, not by clusters (maternity centers).

The intervention group was less educated than the control group. Education is widely known to be associated with diet and BMI [19] and may affect the effect of intervention. Pre-pregnancy BMI, smoking status and education were taken into account in the analyses, but the baseline differences in these variables between the groups may have contributed to the efficacy of the intervention. An important factor is the use of antibiotics, which affects the bacterial flora of the intestine, and therefore the level of lignans in circulation. In our study, there were only a few pregnant women who reported the use of antibiotics, and thus, their use may not have played any significant role. Another factor which may have influenced the finding, is that the control group received dietary and physical activity counseling as a part of the standard maternity care, although not at the same level of intensity as the women in the intervention group. Awareness of being in a trial may have influenced the counseling practices of the control nurses, as well as the health behavior of the control women.

The small sample size was also a limitation of this study. Due to the small number of clinics, the clinic-level variation could not be taken into account by using multilevel analysis. Furthermore, there were more drop-outs in the intervention group than in the control group. Although the drop-out reasons were only partly related to the study, the drop-outs might have been less motivated to change their health behavior. In this pilot study, information on diet was obtained by questionnaires, which have not been validated for pregnant women. Additionally, use of food frequency questionnaire may be a limitation since the groups are not directly comparable. Weight before the pregnancy was self-reported, which may induce some bias. However, weight development during the pregnancy was measured by the nurses, and thus more reliably collected.

The pregnant women in this study were healthy primiparas, who had relatively healthy dietary and physical activity habits. Therefore, there may not have been much room to further improve their diet. However, changes were noticed on diet and serum lignan levels among the women in the intervention group, indicating that the dietary intervention was successful.

It is essential to determine whether this pregnancy intervention has any impact on the offspring's health. In our previous study, none of the newborns in the intervention group had a high birthweight [16] - an indicator of increased susceptibility to obesity and breast cancer, suggesting that increasing dietary intake of fruits, vegetables and fiber benefits the offspring.

We have shown earlier by using animal models that changes in maternal diet during pregnancy alter mammary cancer risk among female offspring [20,21]. Other investigators have reported increased susceptibility to cardiovascular diseases, type 2 diabetes and other adverse health effects in animal models and humans caused by insufficient energy or protein intake during pregnancy [22]. Although we generally associate lignan intake to positive health outcomes [7], nothing is known of their effects on the offspring exposed through a pregnant mother. A recent study in rats indicating that an exposure to multivitamins during pregnancy accelerated the development of obesity among offspring [23] raises concerns regarding the safety of pregnancy supplementations. However, there is no evidence that high dietary intake of fruits, vegetables and fiber during pregnancy has any adverse effects on the health of the offspring. Future studies should increase understanding of lignan effects to offspring.

## Competing interests

The authors declare that they have no competing interests.

## Authors' contributions

RL is principal investigator in the clinical trial study. EK, JR and NS were responsible for data management. AIS is responsible for all chemical analyses. EK, MF and JR conducted statistical analyses. LHC contributed to the article on expertise on nutrition and animal models, and all authors contributed in writing of the paper.

## Supplementary Material

Additional file 1 **Flow chart of the trial**. Flow chart of the cluster-randomized trial showing participating clinics, number of women eligible, randomized and drop-outs. Click here for file
