# Supplementary material for: Effect of dietary intervention on serum lignan levels in pregnant women - a controlled trial
Source: Reprod Health. 2010 Oct 8;7:26. doi: 10.1186/1742-4755-7-26 (PMC2958873; doi:10.1186/1742-4755-7-26)
Supplement: Additional file 1 — Flow chart of the trial. Flow chart of the cluster-randomized trial showing participating clinics, number of women eligible, randomized and drop-outs. [file 1742-4755-7-26-S1.DOC]

Appendix 1. Flow chart of the trial

3 intervention clinics

3 maternity clinics

3 child health clinics

Pregnant women with no earlier deliveries (n=101)

refused to participate

(n=20)

miscarriage or change

of residence (n=5)

Informed consent

(n=69, 73% of eligible)

Drop-outs (n=20, 29%)

miscarriage, n=2

relocation, n=4

pregnancy or health, n=3

life situation, n=4

other reasons, n=5

unknown, n=2

Final number of participants (n=49)

refused to participate

(n=15)

Informed consent

(n=53, 78% of eligible)

Drop-outs (n=5, 9%)

life situation, n=2

unknown, n=2

second pregnancy, n=1

Final number of participants

(n=48)

Eligible (n=94)

Eligible (n=68)

refused to participate

(n=7)

3 control clinics

3 maternity clinics

3 child health clinics

Pregnant women with no earlier deliveries (n=95)

Postpartum primiparas (n=54)

refused to participate

(n=15)

miscarriage or change

of residence (n=4)

Informed consent

(n=63, 77% of eligible)

Drop-outs (n=7, 11%)

relocation, n=3

other reasons, n=4

Final number of participants (n=56)

Informed consent

(n=39, 85% of eligible)

Eligible (n=82)

Eligible (n=46)

Drop-outs (n=2, 5%)

second pregnancy, n=2

Final number of participants (n=37)

Postpartum primiparas (n=73)
